# Parenting stress in the early years – a survey of the impact of breastfeeding and social support for women in Finland and the UK

**DOI:** 10.1186/s12884-022-05010-5

**Published:** 2022-09-10

**Authors:** Vivien Swanson, Leena Hannula

**Affiliations:** 1 Psychology Division, School of Natural Sciences, University of Stirling, Stirling, FK9 4LA UK; 2 Metropolia University of Applied Sciences, FI-00079 Helsinki, Finland

**Keywords:** Postnatal parenting stress, Role strain, Breastfeeding stress, Social support, Self-efficacy, Policy context

## Abstract

**Background:**

Being a new parent can be both joyful and stressful. Parenting stress is associated with poorer health and well-being for parents and infant and increased psychological distress. For new mothers, physical and hormonal changes, expectations of mothering and demands of a new baby may cause additional stress. Breastfeeding is promoted as optimal for maternal and infant health, but can have both positive and negative psychological impacts. Formal and informal social support can offset parenting and breastfeeding stress. Source, content and context of support for new parents are important considerations. This study compares two countries with different parenting and breastfeeding contexts, Finland (more supportive) and the UK (less supportive), investigating the role of breastfeeding stress, self-efficacy and social support as predictors of stress and role strain for new mothers.

**Methods:**

A cross-sectional online survey was completed by 1550 breastfeeding mothers of infants up to 2 years old, recruited via social media platforms in Finland and the UK. Predictors of parenting stress and strain, including demograpic factors, childbirth experiences, breastfeeding and social support were investigated.

**Results:**

We found fewer differences between countries than expected, perhaps due to demographic and contextual differences. Women in Finland reported better childbirth experiences, more positive breastfeeding attitudes, and more self-efficacy than in the UK. Levels of parenting stress were similar in both countries. Women in the UK reported more parental role strain, but fewer breastfeeding stressors. Participants accessed more informal than formal supports, including their partner for parenting and facebook groups and family for breastfeeding. Analysis suggested breastfeeding stress and social support had significant direct effects – respectively increasing and reducing parenting stress and role strain, but no moderating effects of social support suggesting support did not change the relationship between breastfeeding and parenting stress.

**Conclusions:**

Results have important implications for the provision of breastfeeding and parenting support for new mothers. Simple interventions to manage stress for mothers in the postnatal period could be beneficial and are easily delivered by supporters. As shown elsewhere, socio-economic and cultural factors are crucial influences on parenting experiences.

**Supplementary Information:**

The online version contains supplementary material available at 10.1186/s12884-022-05010-5.

## Background

Becoming a mother is a major life transition which can be both joyful and stressful. Health and well-being are important for mother and baby’s health during the early years [1] but difficulties adjusting to the demands of parenting a new baby and the parenting role, including stress and role strain, relationship strains, postnatal depression and anxiety are very prevalent. Parental stress is linked with both anxiety and depression and can have a significant negative impact on parents, child health and family relationships [2–4]. Social disadvantage and poverty are also closely linked with parenting stress and distress [5]. A recent systematic review identified a global prevalence of postnatal depression of 18%, with significant national disparity due to health and wealth inequalities [6]. Social support has a significant protective role for psychological distress. Support from professionals and informal support from partner, friends and family in the postnatal period are all important [7–9].

There is excellent evidence that breastfeeding promotes positive health outcomes for mother and infant [10], but it has potential to both ameliorate and increase parenting stress. There are clear psycho-physiological benefits of lactation in the perinatal period, being associated with maternal calmness, lowered anxiety and reduced stress response [11, 12]. However the relationship between stress/anxiety and breastfeeding is bi-directional. Postpartum anxiety or depression can negatively affect infant feeding outcomes [13] and breastfeeding social pressures. Conversely breastfeeding difficulties may lead to stress and anxiety. Many women who are motivated to initiate breastfeeding report difficulties, particularly in the first few weeks. For some women the reality of breastfeeding is very different from the ideals promoted by health professionals regarding initiation and duration [14]. Where the message ‘breast is best’ is promoted, negative breastfeeding experiences, including breastfeeding pain or discomfort, or social embarrassment can lead to a sense of dissatisfaction or ‘failure’, reducing maternal confidence or self-efficacy [15] and increasing psychological distress.

Psychological models of stress capture this bidirectionality using ‘transactional’ approaches, where the relationship between sources of stress (stressors) and stress outcomes (strains) are influenced (mediated or moderated) by intervening appraisal variables, including self-efficacy (confidence), coping and social support [16]. This approach is helpful as it avoids circularity in using ‘stress’ to refer to both causes (stimuli) and outcomes (responses). Stressors can include short and long-term ‘daily hassles’ as well as long-term (chronic) stress and life events – such as becoming a parent [17]. In this study, we investigated breastfeeding ‘hassles’ or stressors as a predictor of parenting stressors (sources of stress) and role strains (perceived impact of stressors on parental role), including positive and negative aspects of parenting in breastfeeding mothers of infants 2 years and under, considering the role of formal (health service, professional) and informal support from others, (partners, family, friends) as a moderating factor or ‘buffer’ [18]. We aimed to capture different facets of support, including the amount and quality of support, and instrumental (practical) and emotional support.

### The importance of context, Finland and the UK

Policy makers seek to increase breastfeeding initiation and duration to promote better population health. Nevertheless, breastfeeding initiation and maintenance rates meet WHO Guidelines in very few countries internationally. Nordic countries have higher rates of breastfeeding initiation and maintenance than others in Europe [19]. Finland aims to become the ‘model’ breastfeeding country in its national breastfeeding action plan for 2018–2022 [20], and prevalence of any breastfeeding and exclusive breastfeeding has increased over the past 10 years [21]. In contrast, the UK has relatively low and reducing breastfeeding rates of breastfeeding initiation and maintenance [22] with variability between the four UK nations related to different health systems and cultural factors [23]. Table 1 below provides a summary of some key differences in early parenting and breastfeeding provision between Finland and the four UK nations. There are also key differences in breastfeeding policies, promotion and health care, and the wider cultural context whereby social norms in Finland are generally breastfeeding positive. General parenting support, including maternity/paternity leave and childcare provision is also more accessible and less costly in Finland than in the UK overall.

**Table 1:** Breastfeeding rates and formal support for new parents comparing Finland and the UK^1^

	**Finland**	**UK**
**Breastfeeding**		
*Initiation*	98%	81% (2010)
*Any BF*	94% (4 weeks)	55% England, (6 weeks) 68% Scotland 37% Wales 35% N Ireland
*Any BF at 6 mths*	77%	34% England 43% Scotland 27% Wales 13% N Ireland
**Formal health care**		
*Unicef Baby friendly Hospitals*	25% hospitals BFHI certificate 85% hospitals BF outpatient clinic 80% hospitals breastmilk-bank	England; 53% of births in BFHI Scotland; 100% Wales: 86% N Ireland 93%
*Postnatal health care (hospital and community) *	**Hospitals:** 100% Midwives WHO BF course 40% Exclusive BF in hospital 97% Partial BF in hospital **Community:** 97% PH nurses WHO BF course BF outpatient clinics in community	UK – Services with full Baby Friendly Accreditation 60% of maternity services 73% of Health Visiting (Community) services
**Formal parenting support**		
*Maternity/paternity leave*	Maternity leave 105 working days Paternity leave 54 working days	52 weeks maternity leave Pay for 39 weeks 1–2 weeks Paternity leave
*Parental leave*	158 working days	18 weeks ( 90 working days)
*Daycare provision*	Subjective right for public day-care	Variable – linked to receiving Government benefits
*Maternity package*	Baby-Box or cash benefit of €170	England – pilot schemes in some areas Scotland – baby box free for Every baby Wales – pilot schemes in some areas

*Abbreviations*: *WHO* World Health Organisation, *BF* Breastfeeding, *BFHI* Baby Friendly Hospital Initiative, *PH Nurse* Public Health Nurse

^1^ Estimated data, accurate comparisons are difficult due to different data collection processes, time-frames and metrics

### Third sector (voluntary) and peer support groups

Both countries have voluntary organisations in place that support mothers’ breastfeeding. The Finnish Association for Breastfeeding (ITU) offers trained peer support for breastfeeding families in Finland. All volunteers have personal breastfeeding experience and have completed ITU’s breastfeeding training [24].

In the UK, similar support organisations exist, such as the Breastfeeding Network (BfN), which aims to be an independent source of support and information for breastfeeding women and others. The BfN provides training to become a Registered Breastfeeding Helper or Supporter [25]. The National Childbirth Trust and La Leche League of Great Britain also provide training for volunteers and breastfeeding support for mothers. Whereas fathers in Finland report helpful informal support structures [26], support for partners (fathers) in the UK tends to focus on single or separated parents.

### Purpose, aim and scope of current research study

This study aims to examine the role of breastfeeding stress, self-efficacy, and social support as predictors of parenting stressors and strains for mothers of children up to 2 years who are currently breastfeeding their infant. The study compares women in Finland, a country with high breastfeeding rates with those in the UK, where rates are much lower.

Hypotheses:

1. Breastfeeding mothers with young children who exhibit more breastfeeding stress and lower self-efficacy will experience more parenting stressors and strains.
2. Breastfeeding mothers who have more, and more helpful formal and informal social support will record less breastfeeding stress, more self-efficacy, and fewer parenting stressors and strains.
3. Breastfeeding mothers in Finland will receive more positive support and experience less breastfeeding related stress and higher self-efficacy than those in the UK.

## Methods

### Design

This was an observational study using an online cross-sectional survey. A theoretically based survey of breastfeeding mothers with children under 2 years was undertaken in Finland in 2016/7 and a comparison survey completed in 2017 in the UK. In both countries, participants were approached via third sector organisations with a breastfeeding and early parenting support role. Although study recruitment is often carried out via formal health services, we wanted to represent the fact that new mothers may seek out both generic (focused on parenting) and breastfeeding specific support, from both informal and formal sources. These different types of support may have different functions and impacts. Using on-line recruitment methods also allowed us to obtain large comparable samples over a relatively short timespan. Data on stress and support was also considered useful to help participating organisations to plan future services. The survey was carried out by researchers from the University of Stirling and Helsinki Metropolia University and distributed using the ‘Qualtrics’ (Qualtrics XM, 2016) package. Paper copies were available but not requested. Early parenting stressors and strains were primary outcomes. Breastfeeding stress, self-efficacy and breastfeeding and parenting support were main predictors. Demographic information was used for sub-group analysis, including comparison of uniparous and multiparous women.

### Recruitment and participants

The survey was open to mothers of children with at least one child of 2 years or under. Only those currently breastfeeding their youngest child were included in this analysis. It was available in Finnish, Swedish (a second language in Finland) and English. The survey link was circulated via contacts in local breastfeeding groups, and social media, and with their permission, was posted on websites of non-government organisations supporting breastfeeding and early parenting in Finland, including:

- The Finnish Association for Breastfeeding Support [24];
- Folkhalsan; https://www.folkhalsan.fi/en/
- The Federation of Mother and Child Homes and Shelters; https://ensijaturvakotienliitto.fi/en/
- and including UK Breastfeeding Networks [25], the National Childbirth Trust: https://www.nct.org.uk/ and the LaLeche League, https://www.laleche.org.uk/

Respondents in the UK (*n* = 533) were from England (210, 13%), Scotland (306, 20%), Wales (5,1%) and Northern Ireland (12,2%), with 1017 (65%) from Finland.

### Sample size and statistical power

Studies using demographic and psychological variables as predictors of stress generally show a medium effect size using multiple regression analysis. Using the G*Power package [27] for an effect size of r^2^ = 0.3, with power of 0.90 and alpha 0.01 a sample of approximately 102 women in each country was required. To carry out secondary analysis we aimed to recruit substantially more participants in each context.

#### Inclusion/ exclusion criteria

The study included women who had ever breastfed their child of 2 years old or under. This paper reports data from those currently breastfeeding. There were no exclusion criteria, although we were not able to offer translation of the survey into other languages, which may have excluded some women.

### Measures

Validated measures were used where possible. To encourage completion and reduce participant burden, measures were shortened, and where necessary modified to remove items inappropriate for the target population.

### Demographic variables

Maternal age, education level, residence and financial security were proxy variables for socio-economic status and potential predictors of infant feeding distress and general stress [5, 13].

*Parental status:* coded according to status - with partner or single parent.

*Age*: 4 groups from 18–24 to over 50. Group: 1 = 18–24, 2 = 25–34, 3 = 35–49, 4 = 50 and over.

*Education:* Due to some differences in education systems in each country, highest level of education was measured in four categories, scored 1–4: Secondary, Further education (including vocational), University and Postgraduate.

*Residence* was assessed as own (owned) home vs other, and ‘urban’, ‘suburban’, ‘rural’ (recoded as urban/rural).

*Financial security* was assessed on a 6 point scale: ‘Meeting my normal household expenses is..’: from 1 ‘very difficult’ to 6 ‘very easy’.

### Parenting and Infant feeding variables

*Childbirth:* delivery method was coded in categories as vaginal; planned C-section; unplanned C-section.

*Skin-to-skin contac*t immediately after delivery was coded ‘yes/no’.

*Overall birth experience* was rated on a 5 point scale from 1 = very bad to 5 = very good.

*Children:* Number and ages of children living at home, recoded as uniparous/multiparous.

Youngest child’s age was coded as 0–11 months; 12–23 months; and 23 months and over.

*Infant Feeding:* We asked women ‘how are you currently breastfeeding your baby?’ with responses coded as only breastmilk; mixed breastmilk and formula; only formula or ‘does not apply’ for weaned infants (not included in this analysis).

The survey used validated psychological measures for Professional Support [28], and modified measures of Parental Stress [29], Role Strain [30], and Breastfeeding Self-efficacy [31] as described below. Breastfeeding attitudes [9] and shared parenting measures [32] had been used in previous research. Parenting and breastfeeding support measures were developed for the study.

### Attitudes

To compare breastfeeding attitudes we used 7 items from Swanson and Power (2005) relevant to new parents [9]. For example, breastfeeding is: natural, painful, convenient; rated on a 5 point Likert scale from (1) ‘strong disagreement’ to (5) ‘strong agreement’. Three items are reverse scored. Items were summed to create total scores, possible range 7–35. Higher scores represent more positive attitudes to breastfeeding. Cronbach’s α. = 0.71.

### Parenting and breastfeeding stress

#### Parenting stressors

We utilized 8 items from Park et al.’s (2015) 9 item Postpartum Stress Scale [29], to measure sources of stress, including; relationships, being a mother, fussy baby, finances, work, own health, sleep, and health concerns, measured on a 4 point scale (scored 0–3) from ‘not at all stressful’ to ‘very stressful’, score range 0–24. Cronbach’s α. = 0.77. One item measuring ‘breastfeeding stress’ was excluded as we measured this separately.

#### Parenting role strains

Six items relevant to parents of children in this age group were summed, taken from Berry and Jones’ (1995) 18 item Parenting Stress Scale [30]. The measure reflects ‘strains’ including positive and negative outcomes of the parental role. Participants are asked about their experience of being a parent (e.g. ‘Caring for my baby sometimes takes more time and energy than I have to give’). Excluded items were those more relevant for older children included ‘having children has been a financial burden’. Items were rated from ‘strong agreement’ to ‘strong disagreement’ on a 5-point scale (scored 1–5). Positive items (eg ‘I am happy in my role as a parent’) were reversed so higher scores represented more role strain, score range 6–30, Cronbach’s α. = 0.73.

#### Breastfeeding-specific stressors

At the time of the study we could identify no suitable measure of common breastfeeding stressful situations. We developed a scale (8 items, summed) from literature review based on ‘the daily hassles’ approach [33], asking participants to rate the stressfulness of common breastfeeding situations (e.g. insufficient milk, breastfeeding outside of home, using a breast pump) rated from ‘not stressful’ (1) to ‘a lot of stress’ (5), possible score range 8–40. Cronbach’s α. = 0.80.

#### Timing of breastfeeding stress

We asked: ‘At what time point did you experience most stress around breastfeeding?’ Fixed choice time points were based on breastfeeding duration data points from the UK Infant Feeding Survey 2010 [34] from ‘*at birth’* to *‘over 1 year’ (1–10).*

#### Breastfeeding Self-efficacy

(BFSE) was measured using four summed items from the Breastfeeding Self-efficacy Scale Short Form [31], representing general breastfeeding confidence,]: e.g. ‘I am able to determine that my baby is getting enough milk’, ‘I can cope with breastfeeding’, ‘I keep wanting to breastfeed’, ‘I am satisfied with my breastfeeding experience’, rated on a 5 point Likert scale from ‘strong disagreement’ to ‘strong agreement’, possible score range 5–20. Higher scores indicate greater self-efficacy, Cronbach’s α. = 0.80.

#### Social support

This was investigated in relation to their youngest child.

#### Informal Parenting Support

Asked for their main source of support with the youngest child: coded as partner, family, and others (including friends, peer supporter, volunteers), and how helpful this support was? (1 = ‘very unhelpful’ to 5 ‘very helpful’).

#### Shared Parenting

Measured instrumental support from partners, using 7 items adapted from Swanson et al. (2015)[32]. Parents are asked who spends the most time in each activity: ‘getting up in the night to look after the baby’, ‘changing nappies’, ‘feeding the baby’, ‘playing with the baby’, ‘taking the baby out’, ‘soothing the baby’, and ‘babysitting/caring for baby in the daytime’; rated on a 5-point scale from ‘mother does all of the time’ to ‘father does all of the time’, scored from minus 2 to plus 2. Zero represented equal engagement in parenting tasks. Total scores were calculated, (range -14 to + 14). Higher scores represented more partner support, Cronbach’s α. = 0.75.

#### Formal Professional Support

From health and social care professionals was measured using Mercer’s (2004) 10 item CARE (Consultation and Relational Empathy) measure [28]. Participants are asked to think about experiences of parenting their youngest child, and rate statements: ‘The professionals who support me have’: e.g. ‘made me feel at ease, introduced him/herself, explaining his/her position, been friendly and warm towards me, treated me with respect; not cold or abrupt’, on a 5-point scale from ‘strongly agree’ (5) to ‘strongly disagree’ (1), and items summed. Higher scores reflect more support, Cronbach’s α. = 0.94.

#### Breastfeeding Support

Asked *‘*where did you access breastfeeding support for your youngest child?’ multiple responses were possible, scored present (1) or absent (0). Support was categorised as *Formal:* including maternity hospital, and baby clinics; *Informal support*: incuded Facebook support groups; other peer support; doula; family; other. Total formal and informal breastfeeding supports were calculated.

### Ethical issues

#### Incentives for completion

To enhance the response rate and reach of the questionnaire, we offered a small prize draw incentive for completion, using online shopping vouchers of 25 UK pounds or Euros.

Prior to administering the survey we obtained Ethical Approval from the University of  Stirling (Scotland) and The Finnish National Board on Research Integrity (TENK). The main ethical issue was participation in the prize draw which required an email address. Consent for the questionnaire was sought in introductory text and implied by completion. The survey was anonymous including no identifiable data. Analysis took place at the University of Stirling. Data was stored on password-protected computers only accessed by the researchers. To mitigate breastfeeding or parenting distress or for those who lacked social support, we included signposting to support services. Wording of the questionnaire was considered carefully and reviewed by professionals in the field.

### Analysis

Descriptive analysis compared demographics, social support, parenting and breastfeeding variables for participants in Finland and the UK. Effect sizes were reported as Cramers V for chi square (strong association > 0.5) and Cohens d for t-tests. Main analysis compared women in Finland and the UK with sub-group analysis of uniparous and multiparous women. Given the inequality in sample size, where there was significant heterogeneity of variance between these groups, (tested using Levene’s test) Welch’s correction was applied [35]. Following exploratory correlation analysis (Pearson’s r), significant (*p* > 0.05) demographic, breastfeeding-related, and social support variables were entered into linear regression in blocks to predict parenting stressors and role strain. Interaction terms were created by multiplying breastfeeding stress and social support variables to investigate moderation effects of social support, and entered in a final step. Values less than *p* = 0.05 were considered significant. Analyses were conducted using SPSS v27.

## Results

### Demographic data

There were notable demographic differences between participants in Finland and the UK as shown in Table 2. Those in Finland were younger, more likely to be a single parent, and reported less financial security. Differences in home ownership and education level reflect systemic structural differences in both countries.

**Table 2:** Demographic details of sample, comparing UK and Finland

	***Finland*** ***(n* = *1017)*** ***N%***	***UK*** ***(n* = *533)*** ***N%***	**χ**^**2 **^***(df)***	***p***	***ES***^***1***^
*Single parent*	*42 (4.1%)*	*9 (2%)*	*6.3(1)*	*.010*	*V* = *.06*
*Education*					
*Secondary*	*123 (12.1%) *	*51(9.6%)*	*132.9(3)*	< *.001*	*V* = *.29*
*Further Ed*	*239 (23.6%)*	*64 (12.1%)*			
*University*	*391 (38.6%)*	*364 (68.5%)*			
*Postgraduate*	*260 (25.7%)*	*52 (9.8%)*			
*Own home*	*687 (67.7%)*	*428 (80%)*	*26.3(1)*	< *.001*	*V* = *.13*
*Urban*	*692 (68.2%)*	*349(65.2%)*	*1.44(1)*	*.230*	*V* = *.03*
	***Mean (SD) ***	***Mean (SD)***	***t (df)* = **		***d***
*Age group (18–24 to 50* +*)*	*2.1 (.57)*	*2.4 (.54)*	*-10.48(1130.04) *^*2*^	< *.001*	*-.56*
*Financial security*	*4.2 (1.0)*	*4.4 (1.0)*	*-3.57(1548)*	< *.001*	*-.19*

^1 ^Effect size: χ^2^ analysis expressed as Cramers V; t-tests as Cohen’s d

^2 ^Welch’s correction for heterogeneity of variance applied

Uniparous women made up two thirds of participants in both countries, were younger (mean age group 2.1 SD 0.6 vs. mean 2.3 SD.56; t(1475.77) = -5.09, *p* < 0.001, d = -0.59), and reported more financial security (mean 4.3 SD 1.1 vs. mean 4.1 SD1.0; t(1460.334) = 2.86, *p* = 0.002, d = 0.15) than others. Half of participants’ youngest child in both Finland (542,62%) and the UK (420, 63%) was under 1 year. Women in Finland had more children on average as shown in Table 3.

**Table 3:** Women’s child characteristics, childbirth and breastfeeding experience, comparing UK and Finland

	***Finland*** ***N(%)***	***UK*** ***N(%)***	χ^2^ (**df)**	***p***	***ES***^***1***^
*Uniparous*	*581 (57%)*	*299 (56%)*	*.10(1)*	*.755*	*V* = *.01*
*Youngest Child:*					
*0–11 months*	*663 (66%)*	*300 (57%)*			*V* = *.12*
*12–23 months*	*344 (34%)*	*217 (41%)*	*23.6 (2)*	< *.001*	
> *24 months*	*2 (0.2%)*	*11 (2.1%)*			
*Delivery:*					
*Vaginal*	*872(86%)*	*396 (74%)*			
*Planned C section*	*49(4.8%)*	*48 (9.0%)*	*33.3(2)*	< *.001*	*V* = *.15*
*Unplanned C section*	*94 (9.3%)*	*91 (17.0%)*			
*Skin to skin:*	*855 (84%)*	*416 (78%)*	*10.0 (1)*	*.002*	*V* = *.08*
*Current feeding*					
*Only breastmilk*	*878 (87%)*	*456 (85%)*			
*Mixed*	*137 (13.5%)*	*77 (14%)*	.*26 (1)*	*.607*	*V* = *.01*
	***Mean (SD)***	***Mean (SD) ***	***t (df)***		***d***
*No of children (range 1–6)*	*1.6 (.94)*	*1.5 (.71)*	*-10.647 (1360.86)*^*2*^	*.024*	*.11*
*Overall birth experience: 1 v bad – 5 v good)*	*3.9 (1.1)*	*1.7 (0.62)*	*49.187 (1544.6)*^*2*^	< *.001*	*2.22*
*Breastfeeding attitudes*	*31.9 (3.1)*	*29.2 (2.9)*	*16.237(1039.91)*^*2*^	< *.001*	*.87*

^1 ^Effect size: χ^2^ analysis expressed as Cramers V; t-tests as Cohen’s d

^2 ^Welch’s correction for heterogeneity of variance applied

More women in Finland had vaginal delivery, whereas more in the UK had planned or unplanned C Section. Skin-to-skin experience post delivery was more common in Finland, where women also reported significantly more positive childbirth experiences overall. Most women in Finland and the UK were currently feeding only breastmilk, rather than breastmilk and formula. Women in Finland also had more positive attitudes to breastfeeding.

### Parenting and breastfeeding stress

Parenting stressors and role strains were assessed. There was no difference between women in Finland and the UK in level of parenting stressors, but women in the UK reported more parenting role-strain than those in Finland, as shown in Table 4. Women in Finland reported more breastfeeding stressors, and higher breastfeeding self-efficacy than those in the UK.

**Table 4:** Comparison of parenting stressors, role strains, breastfeeding stress and self-efficacy for participants in Finland and the UK

	***Finland*** ***Mean (SD)***	***UK*** ***Mean (SD)***	***t (df)***	p	***ES***^***1***^
*Parenting Stressors*	*9.9 (4.4)*	*9.6 (4.3)*	*1.33 (1164)*	.092	.08
*Parenting Role Strain*	*13.8 (3.8)*	*16.7 (3.4)*	*-15.32(1184.33)*^*2*^	< .001	-.79
*Breastfeeding Stressors*	*15.0 (5.2)*	*14.3(4.2)*	*2.75(965.88) *^*2*^	.003	.16
*Breastfeeding self-efficacy*	*18.0 (2.5)*	*17.3 (2.7)*	*4.65(835.49)*^*2*^	< .001	.27

^1 ^Effect size: Cohen’s d

^2 ^Welch’s correction for heterogeneity of variance applied

Uniparous women reported lower breastfeeding self-efficacy than others (mean 21.8 SD3.1 vs. mean 22.5 SD2.8; t(1401.32) = -4.49, *p* < 0.001, d = -0.24). However there was no difference in parenting stressors or strains, or breastfeeding stress in relation to parity.

Women with better overall birth experience reported higher self-efficacy (*r* = 0.16, *p* < 0.001), and less parental role strain (*r* = -0.36, *p* < 0.001), but there was no relationship with parenting stressors or breastfeeding stress.

**Fig. 1:**
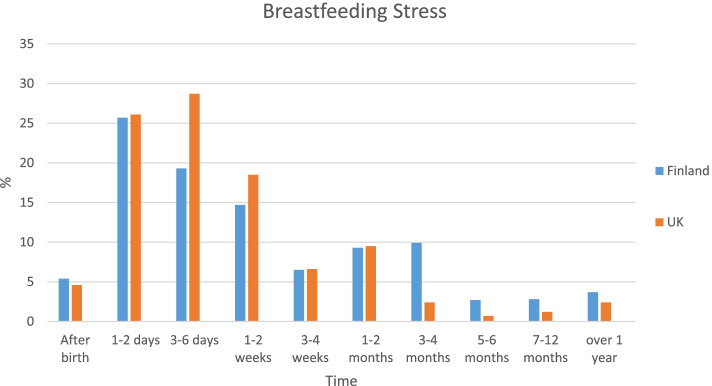
Comparison of the most stressful periods during breastfeeding for women in Finland and the UK

Figure 1 illustrates which periods women reported experiencing most breastfeeding stress, comparing Finland and the UK. The most stressful breastfeeding time for all women was the week after childbirth, reflecting hormonal changes and learning to breastfeed. There was a significant difference between women in Finland and the UK in the distribution of stress reported (χ^2^(9) = 42.45, *p* < 0.001, V = 0.18), with observed significant differences at 3–6 days and 3–4 months post childbirth.

### Parenting support

Women’s main parental supporter in both countries was their partner, followed by family, friends and peers, as shown in Table 5. Their support was rated as very helpful overall, albeit more positively by UK participants. In the whole sample, there was no difference in ‘helpfulness’ ratings for partners (mean = 4.7; SD.54), family (mean = 4.8; SD, 0.43) or friends ((mean 4.7; SD.57), F(2,1533) = 0.58, *p* = 0.558).

**Table 5:** Social support for parenting and breastfeeding

**Parenting support**					
**Main supporter**	**Finland** **N(%)**	**UK** **N(%)**	**χ**^**2**^**(df)**	***p***	**ES**^***1***^
Partner	883 (82.1%)	463(86.5%)	10.0 (3)	.018	V = .08
Family	118 (11.6%)	51(9.5%)			
Friends, peers	51 (5.0%)	21(3.9%)			
None	13 (1.3%)	0 (0%)			
	**Mean (SD)**	**Mean (SD)**	**t (df)**	**p**	**d**
How helpful (main supporter)?	4.8 (.56)	4.6 (.57)	4.85 (1050.13)^*2*^	< .001	.26
Shared parenting (range -14 to + 14)	-4.7 (3.1)	-5.4 (3.0)	4.67 (1483)	< .001	.25
Quality of Professional Support (CARE)	35.7 (7.6)	36.5 (3.1)	-2.19 (976.02)^*2*^	.029	-.12
**Breastfeeding Support**					
Number of formal supports	.65 (.77)	.68 (.72)	-.81 (1154.08)^*2*^	.418	-.04
**Formal Support**	**N(%)**	**N(%)**	**χ**^**2**^**(1)**	**p**	**V**
(Health services)					
Hospital	397 (39.1%)	229 (42.8%)	1.98	.159	.04
Baby Clinics	263 (25.9%)	136 (25.4%)	.044	.834	.05
Number of informal supports	1.1 (.92)	1.5 (.68)	-7.39 (944.60)^*2*^	< .001	-.41
**Informal support**					
Facebook Group	551 (54.3%)	272 (50.8%)	1.7	.196	-.03
Other peer suppt	75 (7.4%)	164 (30.7%)	145.3	< .001	.31
Doula	17 (1.7%)	8 (1.5%)	0.07	.790	.01
Family	392 (38.6%)	249 (46.5%)	9.1	.003	.08
Other	86 (8.5%)	116 (21.7%)	53.9	< .001	.19

^1 ^Effect size: χ^2^ analysis expressed as Cramers V; t-tests as Cohen’s d

^2 ^Welch’s correction for heterogeneity of variance applied

Mothers in Finland had less negative scores in relation to shared parenting, although mothers still did more of each task than their partners. Considering individual items in this measure, partners of women in Finland were more likely to support getting up in the night (*p* < 0.001) feeding baby (*p* < 0.001), playing with baby (*p* = 0.01), and soothing baby (*p* = 0.009), than those in the UK. Women in Finland rated general professional support (empathy) slightly lower than those in the UK.

### Breastfeeding support

Overall, women used much more informal parenting support than formal hospital and baby clinic support for breastfeeding as shown in Table 5. This is unsurprising since formal health service support tends to focus on the immediate postnatal period.

Uniparous women reported more shared parenting (mean -4.9; SD2.9, vs mean -5.6; SD3.0; t(1478) = 3.95, *p* < 0.001, d = 0.21) than other women.

There was no difference in reported quality of professional support (CARE) for uniparous compared with multiparous women.

UK women used more informal supports overall for breastfeeding than those in Finland with no difference in formal breastfeeding support. The most common sources of breastfeeding support were Facebook peer support followed by family support for both groups. ‘Other’ support included national support organizations which were more common in the UK.

### Predicting parenting stressors and strains

Two sets of multiple linear regression analysis were carried out, (Table 6) predicting parenting stressors and role strain from breastfeeding stress and social support for the whole sample. Demographic factors, breastfeeding, social support and interactions were entered in steps.

**Table 6:** Multivariate regression analyses predicting parenting stressors and parental role strain for breastfeeding women in Finland and the UK

	**Parenting stress**			**Parental role strain**		
**Model 1 Demographics**	**β**^***1***^	**p**	**(95%CI)**	**β**^***1***^	**p**	**(95%CI)**
Country (Finland/UK)	-.03	.341	(-.88,.30)	.37	< .001	(2.6,3.6)
Single parent	-.04	.170	(-5.1,.91)	-.03	.342	(-2.8,-0.9)
Income	-.28	< .001	(-1.5,-.92)	-.10	< .001	(-.58, -.17)
Education	.06	.040	(.06,.53)	.05	.005	(.01,.47)
R^*2*^_adj_. = 10, F(4,857) = 27.77, *p* < .001				R^*2*^_adj_ = .14, F(4,,1110) = 50.4, p < .001		
**Model 2 Breastfeeding**						
Breastfeeding stress	.11	.004	(.03,.16)	.20	< .001	(.11,.21)
Breastfeeding self-efficacy	-.07	.065	(-.22,.03)	-.07	.040	(-.17, -.01)
R^*2*^_adj_ = .13, F (2,855) = 15.72, *p* < .001				R^*2*^_adj_ = .21, F(2,1108) = 43.9, *p* < .001		
**Model 3 Social Support**						
Professional support (CARE)	-.15	< .001	(-.12,-.05)	-.08	< .005	(-.06, -.01)
Shared parenting	.01	.903	(-.09,.10)	.02	.604	(-.06,.09)
How helpful	-.15	< .001	(-1.6 -.64)	-.10	< .001	(-1.1,-.32)
R^*2*^_adj_ = .17, F(3,852) = 15.73, *p* < .001				R^*2*^_adj_ = .23, F (3,1105) = 7.6, *p* < .001		
**Model 4 Interactions**	**β**^***2***^			**β**^**2**^		
BFStress x CARE	-.23	.162	(-.01,.002)	.01	.870	(-.006, .006)
BFStress x helpful	.12	.710	(-.09,.13)	.37	.172	(-.02,14)
R^*2*^_adj_ = .17, F (2,850) = 1.0, p = .364				R^*2*^_adj_ = .23,F(2,1103) = .94, p = .395		

^1 ^Coefficients reported from Model 3

^2 ^Coefficients reported from Model 4

For parenting stressors, 17% of variance was predicted in the final model. Less financial stability (income) and more education, but not country (Finland, UK) predicted more parenting stress. More breastfeeding stress predicted greater parenting stress. More professional empathic support (CARE), and more overall helpfulness of parenting support were related to lower parenting stress but shared parenting support was not.

A larger proportion of variance (23%) was predicted for parental role strain. In this model, being a UK participant, less financial security and more education were significant predictors of increased role strain. Breastfeeding stress predicted more role strain, whereas higher self-efficacy, more professional support and helpful support predicted lower role strain.

### Moderation effects

To investigate the role of social support as a moderator or ‘buffer’ of the impact of breastfeeding stress on parenting stress, interaction terms for significant support variables were added for parenting stressors and parenting role stressors. Interactions between support and breastfeeding stress did not add to prediction of parenting stress or parenting role strain in regressions, suggesting support did not influence the strength of relationship between both types of stress.

## Discussion and conclusions

This was a large-scale, novel survey of parenting and breastfeeding stress, including uniparous and multiparous breastfeeding mothers across the socio-demographic spectrum from Finland and the UK. These two northern European countries have different formal support systems and different cultural norms related to early parenting and breastfeeding. We identified fewer differences than expected between these countries in terms of stress and perceptions of support, reflecting the complexity of different individual factors in different cultural contexts.

Becoming a new parent is both a rewarding and stressful life event [17]. It is important for maternal and infant wellbeing to be aware of potential sources of postnatal parenting stress and strain, and to offer appropriate and helpful care and support when needed. This study found breastfeeding-related stress was a signicant predictor of increased parenting stressors and role strain. It is important to consider potential mechanisms for this relationship. Our study was cross-sectional, so we cannot infer causality from our data. It may be that women who are experiencing more parenting stress and role strain related to their living circumstances, health or relationship problems or other stressors find breastfeeding behaviours more challenging. Conversely, the challenges of breastfeeding may exacerbate existing parenting problems. Successful breastfeeding, with regular oxytocin release may make it easier to cope with everyday hassles [11, 12]. It would be helpful to consider the relationship between postnatal stress, anxiety and depression, breastfeeding and parenting factors in a longitudinal design, developing the work of Law et al. (2019) [36] in larger samples, and considering interactions between women and their partners in this context. Developing confidence in both breastfeeding and parenting is clearly an important method of reducing stress and breastfeeding self-efficacy was related to less parenting stress in our study as elsewhere [36]. It is interesting to note that the trajectory of reported breastfeeding stress in the postpartum period – with higher stress in the early weeks and 5–6 month period was also similar in the current study to that reported elsewhere in uniparous women [36].

Much research into breastfeeding understandably focuses on the health benefits for mother and infant, including the positive psychological impact of breastfeeding and breastmilk on mental wellbeing [11, 37]. However, it is important to consider wider impacts of breastfeeding in different social contexts and possible negative stressors and strains on parenting, family, and mother-infant relationships. Studies which do consider negative psychological impacts generally focus on clinical levels of anxiety and depression, rather than stress. Nevertheless, stress is much more ubiquitous and can be a precursor to clinical psychological distress. Interventions to reduce stress such as relaxation and mindfulness are prevalent and effective, and can be easily delivered by health professionals. Levels of parenting stressors and strains in this study overall were moderate, with mean scores around 10/24 and 15/30 respectively. Stress is a multidimensional construct and reliable measurement of stress is challenging [38]. We focused on both parenting ‘stressors’ and ‘strains’ to capture sources and outcomes, but were not able to directly compare stress levels in our sample with other work. Future work could focus on establishing population or normative ‘benchmarks’ to direct interventions, and on investigating relationships between postnatal stress, anxiety and depression in more depth [39].

As in other studies, we found a positive direct relationship between social support and reducing parenting and breastfeeding stress [7, 8]. Formal support (professional care and empthy) and helpful informal parenting support were both related to reduced parenting stressors and strains. Different systems of health service support in Finland and the UK in the postnatal period may have influenced participants’ expectations and perception of the empathic care they received in hospital, baby clinics and the community.

Informal support was used much more than formal support. Both ‘instrumental’ support, focused on specific stressors such as worries about the baby, sleep problems and loneliness, and ‘emotional’ support for the parental role are important. Expectations of ‘ideal’ parenting roles increasingly appear to emphasise the importance of ‘shared parenting’ [32]. Practical shared parenting (e.g. babysitting, getting up in the night) was not a predictor of reduced stress in this study, although it was notable that partners were described as the main source of parenting support for most participants, despite differences between Finland and the UK in parental leave policies. Further research could fruitfully explore qualitative differences in gender roles and support, comparing countries with different systems. Quality rather than quantity of supportive relationships is clearly important. Positive interactions between partners around parenting and breastfeeding in the postnatal period may reduce psychological distress and negative long-term health outcomes for children and families [40].

In relation to breastfeeding support, over half of women in Finland and the UK described Facebook groups as their main resource, followed by families. Others have noted the increased prevalence and value of social media support for breastfeeding [41, 42], suggesting the quality and effectiveness and timing of this type of informal support matters — online groups can provide immediate access to advice that may be difficult to obtain ‘in person’. However the quality of social media support and advice is variable, depending on the credibility of the source and quality of information presented, and could have negative rather than positive impacts on well-being. Of course, this may also be the case for other formal and informal sources of support.

We anticipated less parenting and breastfeeding stress in Finland, reflecting the country’s more positive and supportive policies, norms and attitudes [20, 21, 43]. Both countries focus significant policies and resources on supporting new parents, and breastfeeding, however the UK has lower breastfeeding rates, a larger, more diverse population, higher overall levels of deprivation, and more regional variation in services. Women in Finland reported more shared parenting, more positive attitudes to breastfeeding, and higher breastfeeding self-efficacy than those in the UK as expected. Their childbirth experience, often an indicator of psychological distress [44] was more positive. However we found no difference in parenting stressors. UK women reported more parenting role-strain than those in Finland, but women in Finland reported more breastfeeding stressors, which was somewhat unexpected. We can speculate that there may be more perceived social pressure and higher expectations regarding exclusive breastfeeding in Finland, which is an additional source of stress. Alternatively this finding may reflect the ubiquity of ‘hassles’ related to integrating breastfeeding into daily life for new mothers, their unrealistic expectations of breastfeeding as being ‘easy’ [14] and the demographic characteristics of the samples – women in Finland were younger, and reported less financial security than those in the UK, being potentially more vulnerable to stress.

### Weaknesses

This was a cross-sectional study, with unbalanced demographic characteristics. We did not collect data on ethnic differences which may have had important implications for women’s experience of stress. It is also important to acknowledge that many families experience additional issues, for example pre-term birth, or caring for children with special needs, which may also influence their psychological wellbeing. These were unfortunately not specifically covered in this study. Acknowledging that new parents experience significant time pressure, we modified some previously validated measures used, reducing the number of items to increase acceptability and completion rates, and to reduce participant burden, but this may have reduced reliability in measurement. There was also unbalanced sampling in numbers, and unequal variance in some variables, which may have influenced the results by violating assumptions of some tests. Nevertheless, the same pattern of statistical significance was observed following adjustment for hetereogeneity of variance where this was an issue, which provides some reassurance. The large number of statistical comparisons also increases the risk of type 1 error, so results should be treated with caution. Participants were recruited ‘informally’ rather than via health service routes, to obtain socially diverse samples, however as with many surveys, it proved difficult to achieve this. It is likely that the larger sample in Finland offers a more representative picture of the overall population of breastfeeding women than the smaller, less-representative sample in the UK. Finally, the study did not cover the impact of specific potential breastfeeding stressors, such as exclusive breastfeeding, expressing and storing breastmilk or combining breastfeeding with employment or other roles. These are important future research questions.

## Conclusions

Information provided from this study highlights the potential for stress in new parents, and potential links between breastfeeding and stress. Stress is relatively easily and economically addressed at scale. Future research could develop and trial the implementation of simple stress management interventions for new parents to prevent more serious psychological distress. Informal and formal organisations providing support for breastfeeding could use this information to support new parents more effectively, promoting greater breastfeeding satisfaction and enhancing psychological well-being in new parents.

## Supplementary Information

**Additional file 1. **

## Data Availability

All data generated or analysed during this study are included in this published article [and its supplementary information files].
